# Differential Responsiveness of Cortical Microtubule Orientation to Suppression of Cell Expansion among the Developmental Zones of *Arabidopsis thaliana* Root Apex

**DOI:** 10.1371/journal.pone.0082442

**Published:** 2013-12-04

**Authors:** Emmanuel Panteris, Ioannis-Dimosthenis S. Adamakis, Gerasimos Daras, Polydefkis Hatzopoulos, Stamatis Rigas

**Affiliations:** 1 Department of Botany, School of Biology, Aristotle University of Thessaloniki, Thessaloniki, Macedonia, Greece; 2 Department of Biotechnology, Agricultural University of Athens, Athens, Greece; Wuhan University, China

## Abstract

Τhe bidirectional relationship between cortical microtubule orientation and cell wall structure has been extensively studied in elongating cells. Nevertheless, the possible interplay between microtubules and cell wall elements in meristematic cells still remains elusive. Herein, the impact of cellulose synthesis inhibition and suppressed cell elongation on cortical microtubule orientation was assessed throughout the developmental zones of *Arabidopsis thaliana* root apex by whole-mount tubulin immunolabeling and confocal microscopy. Apart from the wild-type, *thanatos* and *pom2-4* mutants of *Cellulose SynthaseA3* and *Cellulose Synthase Interacting1*, respectively, were studied. Pharmacological and mechanical approaches inhibiting cell expansion were also applied. Cortical microtubules of untreated wild-type roots were predominantly transverse in the meristematic, transition and elongation root zones. Cellulose-deficient mutants, chemical inhibition of cell expansion, or growth in soil resulted in microtubule reorientation in the elongation zone, wherein cell length was significantly decreased. Combinatorial genetic and chemical suppression of cell expansion extended microtubule reorientation to the transition zone. According to the results, transverse cortical microtubule orientation is established in the meristematic root zone, persisting upon inhibition of cell expansion. Microtubule reorientation in the elongation zone could be attributed to conditional suppression of cell elongation. The differential responsiveness of microtubule orientation to genetic and environmental cues is most likely associated with distinct biophysical traits of the cells among each developmental root zone.

## Introduction

The root is a plant organ growing axially due to highly anisotropic cell expansion. Root cells originate from the mitotically active meristem and progressively elongate parallel to the root axis. This elongation pattern is imposed by cell wall reinforcement that prevents growth in any other direction. Cellulose microfibrils in root cell walls are strictly transverse to the root axis [1], providing the mechanical reinforcement for root morphogenesis. 

Cortical microtubule organization modulates cell wall pattern in various cell types to support plant morphogenesis [2-5]. The alignment hypothesis [6] suggests that cortical microtubules guide the movement of cellulose synthase complexes that produce cellulose microfibrils [7]. In rapidly growing root cells of the elongation zone, cortical microtubules run parallel to cellulose microfibrils [1]. Furthermore, live cell imaging experiments in hypocotyl cells provided evidence that cortical microtubules guide the movement of cellulose synthase complexes [8-11]. Cortical microtubules are connected to cellulose synthase complexes via the Cellulose Synthase Interacting1 (CSI1) protein, supporting the role of microtubules in cellulose microfibril orientation [12-16]. Cortical microtubules may also influence cell wall properties by regulating cellulose microfibril length [17,18] or crystallinity [19-21]. 

On the other hand, cell wall influences the microtubule pattern. In tobacco protoplasts, a biophysical feedback from the cell wall has been reported to mediate transverse orientation of cortical microtubules [22]. Genetic analysis of *Cellulose Synthase A* (*CesA*) mutants and experiments with cellulose synthesis inhibitors revealed that CesA impairment and cellulose deficiency disturb cortical microtubule stability and orientation [23-26]. Consequently, there is a fundamental interplay between cortical microtubule orientation and cell wall integrity [27]. 

The orientation of cortical microtubules in meristematic root cells of *Arabidopsis thaliana* remains ambiguous. Cortical microtubules in interphase meristematic cells have been reported to be transverse [28-30]. However, other reports showed that microtubule orientation was variable [1], random [31], or net-like [32]. In addition, the effects of cellulose biosynthesis or cell expansion inhibition on microtubule organization in the meristematic zone have not been studied yet.

In this study, *A. thaliana* wild-type, *thanatos* (*than*) and *pom2-4* mutants of *CesA3* [33] and *CSI1* [14], respectively, were examined for cortical microtubule organization in root tips. *CesA3* is expressed throughout expanding tissues with primary cell walls and *CSI1* is co-expressed with *CesA3* [12]. The effects of chemical compounds and growth in soil on microtubule orientation were also assessed, to dissect the effect of defective cellulose synthesis or inhibition of cell expansion. Our results support the view that transverse cortical microtubule orientation in *A. thaliana* root tip is established early in the meristem. Furthermore, suppression of cell expansion caused by genetic, chemical and mechanical approaches was associated with microtubule reorientation in the elongation zone, whereas the transverse orientation remained constant in the meristematic zone. 

## Materials and Methods

### Plant material and growth conditions

*Arabidopsis thaliana* wild-type (ecotype Col-0), *than* and *pom2-4* seeds were surface sterilized and kept in the dark at 4°C for 72 h. Seeds were germinated on modified Hoagland’s solution (2 mM KNO_3_, 5 mM Ca[NO_3_]_2_, 2 mM MgSO_4_, 2 mM KH_2_PO_4_, 0.09 mM Fe-EDTA) supplemented with 2% (w/v) sucrose and solidified with 1% (w/v) phytoagar (Duchefa, Haarlem, the Netherlands). Seedlings were grown in Petri dishes with 10 ml of medium, placed vertically in a growth chamber at 21 ± 1°C with a cycle of 16 h light/8 h dark and light intensity of 120 µmol m^–2^ s^–1^. For soil experiments, seeds were sown in soil pots and grown for 5-7 days in the chamber. 

### Chemical treatments

Wild-type seedlings, 5-7 days after germination in Petri dishes, were subjected to the following treatments. Isoxaben (Sigma-Aldrich, Steinheim, Germany) was diluted from 10 μM stock solution in DMSO to a final concentration of 100 nM and was applied for 4 h or 6 h. Aqueous solution of 5 mg/L Congo red (G. Grübler & Co., Berlin, Germany) was freshly prepared and applied for 6 h. Combined application of 100 nM isoxaben and 5 mg/L Congo red was performed for 6 h. Cytochalasin-B (Applichem, Darmstadt, Germany) was diluted from 10 mM stock solution in DMSO at final concentration of 20 μΜ and was applied for 6 h. Aqueous solution of 20 mM 2,3-butanedione monoxime (BDM; Sigma-Aldrich, Steinheim, Germany) was freshly prepared and applied for 6 h. Heterozygous *than*/+ and homozygous *pom2-4* seedlings were treated for 4 h with 100 nm isoxaben or for 6 h with 5 mg/L Congo red. Treatments were performed at room temperature, by pouring 5 ml of each chemical compound solution over the seedlings inside the Petri dish, while the dish was continuously shaken on a rocking platform. In the control samples, seedlings were treated as above with 1% DMSO (for isoxaben), 0.2% DMSO (for cytochalasin-B) or water (for Congo red and BDM). 

### Immunolocalization and angle measurements

Seedlings were prepared for whole-mount immunofluorescence microscopy as previously described [34], with both anti-*α*-tubulin (YOL1/34, AbD Serotec, Kidlington, UK) and FITC-anti-rat secondary antibody (Invitrogen, Carlsbad, CA) diluted at 1:40. The specimens were examined with a Nikon D-Eclipse C1 CLSM (Nikon, Tokyo, Japan) using the default filter set. Digital images were processed with Adobe Photoshop CS2 with only linear settings. To analyze the orientation of cortical microtubules in relation to the root axis, we considered that transverse microtubules have an angle of 0°, while longitudinal microtubules have an angle of 90° [35]. Angle measurements were performed in cortex cells located 20-50 μm over the quiescent center (meristem), protodermal cells just beneath the end of lateral root cap (transition zone) and epidermal cells before root hair bulging (elongation zone). Altogether ~40 cells of meristematic, ~15 cells of transition and ~10 cells of fast elongation zone were examined from at least 4 roots in each sample.

### Morphometric analysis

Wild-type, *than*/+ and *pom2-4* 5-day-old seedlings were treated for 6 h with chemical compounds for morphometric analysis. The LEH (length of the first epidermal cell with visible root hair bulge; [36]) and the length of the previous epidermal cell in the elongation zone of the primary root were examined with an Olympus BX-50 light microscope equipped with a DP71 camera, using Cell^^^A (Olympus Soft Imaging Solutions). Morphometric data were derived from digital images using the ImageJ software package (http://rsb.info.nih.gov/ij/). Measurements of 30 cells in each case were statistically processed with Microsoft Office Excel 2007. 

## Results

### Cortical microtubule orientation is predominantly transverse in wild-type root-tip cells

**Figure 1:**
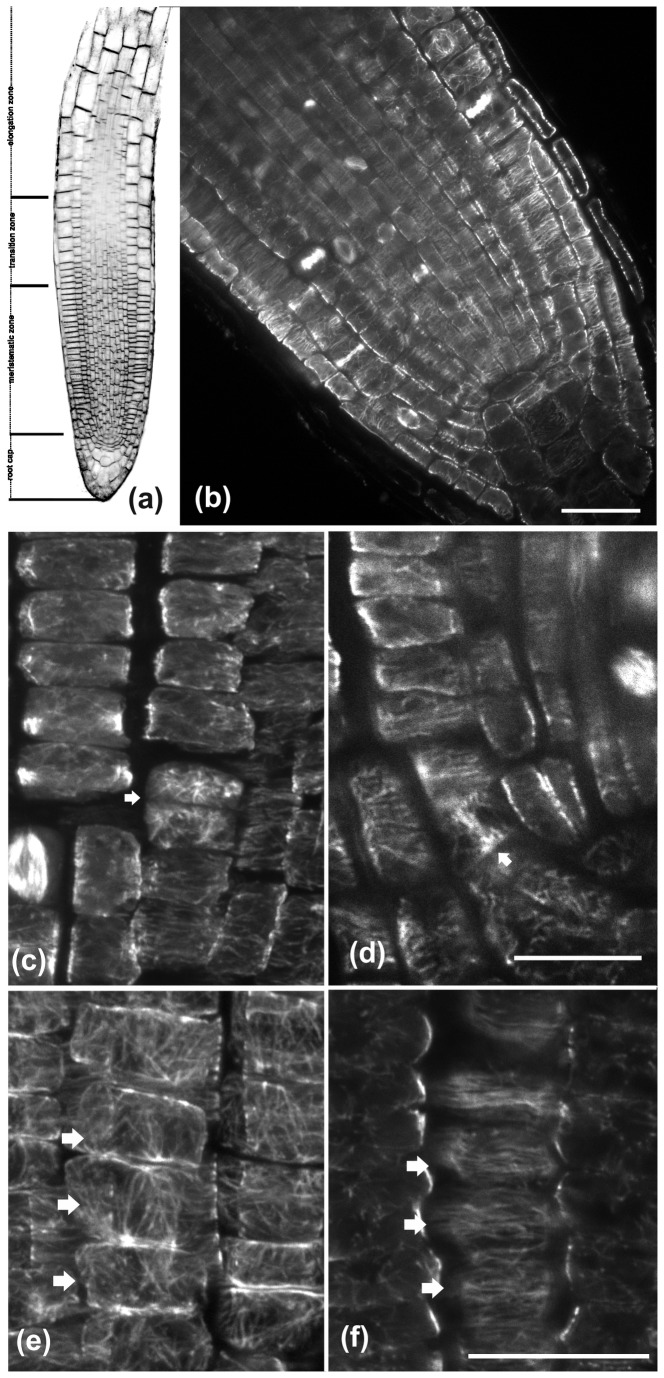
CLSM images of wild-type *A. thaliana* roots after staining with FM4-64 (a) or after tubulin immunostaining (b-f). (a) CLSM longitudinal section through the root tip, illustrating the apical root zones. (b) Cortical microtubules are predominantly transverse in interphase cells of the meristematic zone. (c) In cells that have just divided (arrow) microtubules are randomly oriented. (d) The arrow points a cell close to the quiescent center, dividing almost parallel to the root axis. (e, f) Microtubule organization in protodermal cells of the meristematic zone (marked by arrows). (e) Maximum projection of CLSM sections of the external periclinal cell face. Cortical microtubules exhibit a loose longitudinal orientation. (f) CLSM section through the inner periclinal cell face, where cortical microtubules are transverse. The root tip in these images as well as in the following ones is located towards the bottom of the page. Scale bars, 20 μm.

In this study, the classification of the *A. thaliana* root apex into four zones, the meristematic, transition, fast elongation and growth terminating zone [37], was adopted for analytical purposes (Figure 1a). Lateral root cap covers the cells of the first two zones. The terms “rootward” and “shootward” [38] were also adopted to describe cell location and polarity. 

**Figure 2:**
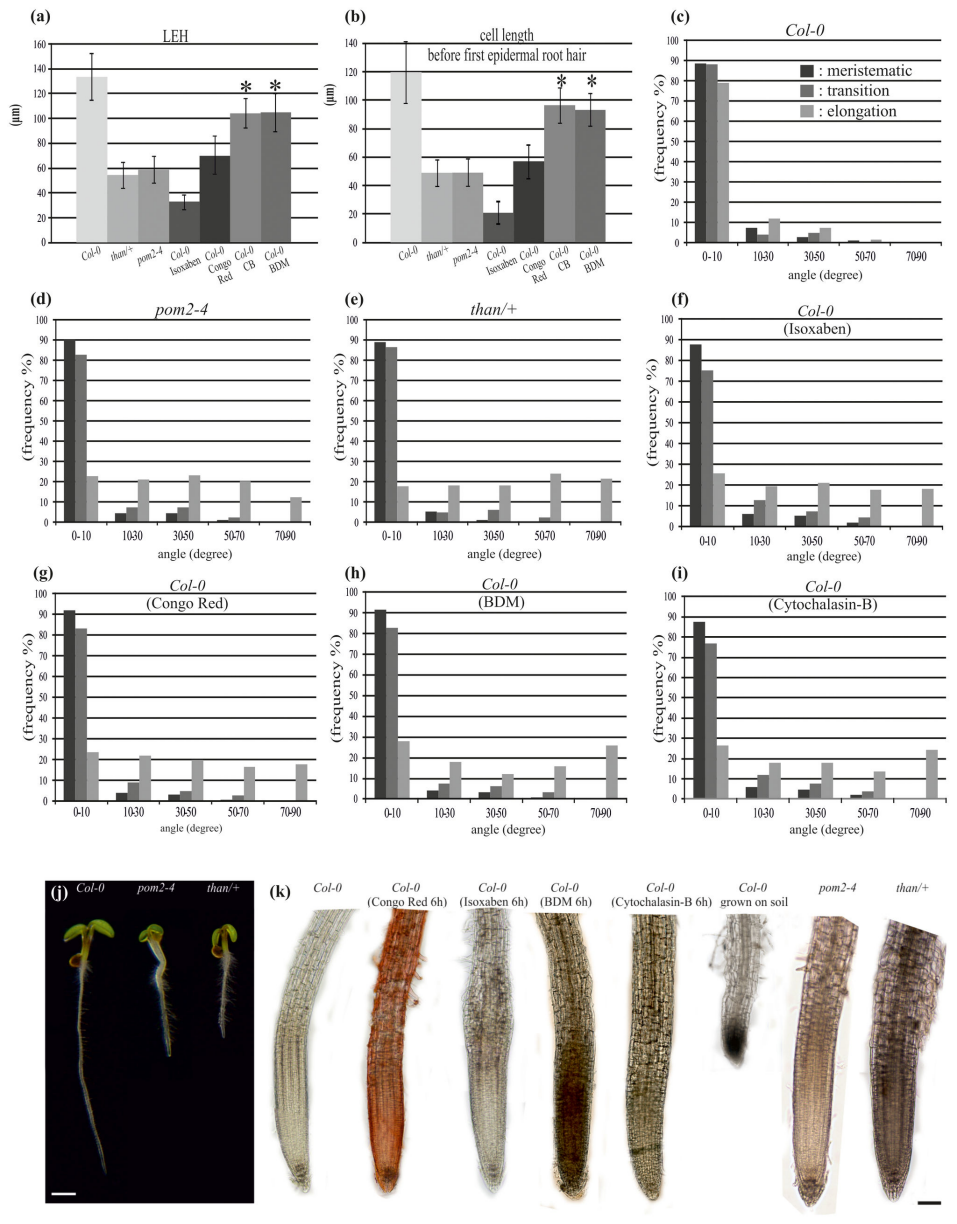
Reduced cell length and modified microtubule orientation in cellulose-deficient mutant and drug-treated roots (a-i). Genetic, chemical and mechanical inhibition of *A*. *thaliana* root growth (j-k). (a) Length of the hair-initiating trichoblast (LEH). (b) Length of the cell before the first epidermal cell with visible root hair bulge. (c-i) Cortical microtubule orientation relatively to the root axis in the developmental zones of untreated wild-type (c), *pom2-4* (d) and *than/*+ (e) primary roots, and of wild-type primary roots treated for 6 h with 100 nM isoxaben (f), 5 mg/L Congo red (g), 20 mM BDM (h) and 20 μM cytochalasin-B (i). *Significant differences compared to the wild-type (P < 0.01). Error bars represent standard deviations of the means. (j) Developmental phenotypes of 5-day-old Col-0 and cellulose synthesis-defective *pom2-4* and *than/*+ seedlings, grown vertically on Petri dishes. (k) Effects of chemical treatments, genetic defects and mechanical impedance on root growth. Chemical compounds were applied at concentrations shown in Materials and Methods. Scale bars, (a) 1 mm, (b) 100 μm.

In the meristematic zone of wild-type roots, cortical microtubule orientation was predominantly transverse, perpendicular to the root axis (Figures 1b, 2c). Nevertheless, meticulous observations revealed three exceptions. First, cortical microtubules exhibited random orientation in cells that had just accomplished cell division (Figure 1c, pointed by arrow). Second, in cells preparing for formative divisions, either periclinal or tangentially anticlinal, cortical microtubules were transverse to the growth axis of each cell but not to the root axis (Figure 1d, arrow). Third, cortical microtubules under the external protodermal cell wall exhibited a loose longitudinal (i.e. parallel to the root axis) orientation (Figure 1e, arrows) similar to the pattern reported previously [39]. However, in these cells cortical microtubules under the radial anticlinal walls and the inner periclinal wall were transversely oriented, perpendicular to the root axis (Figures 1f, pointed by arrows). Apart from these deviations, microtubules were transverse in the inner cell files of the meristematic root zone, including the cortex, endodermis and stele (Figures 1b). 

**Figure 3:**
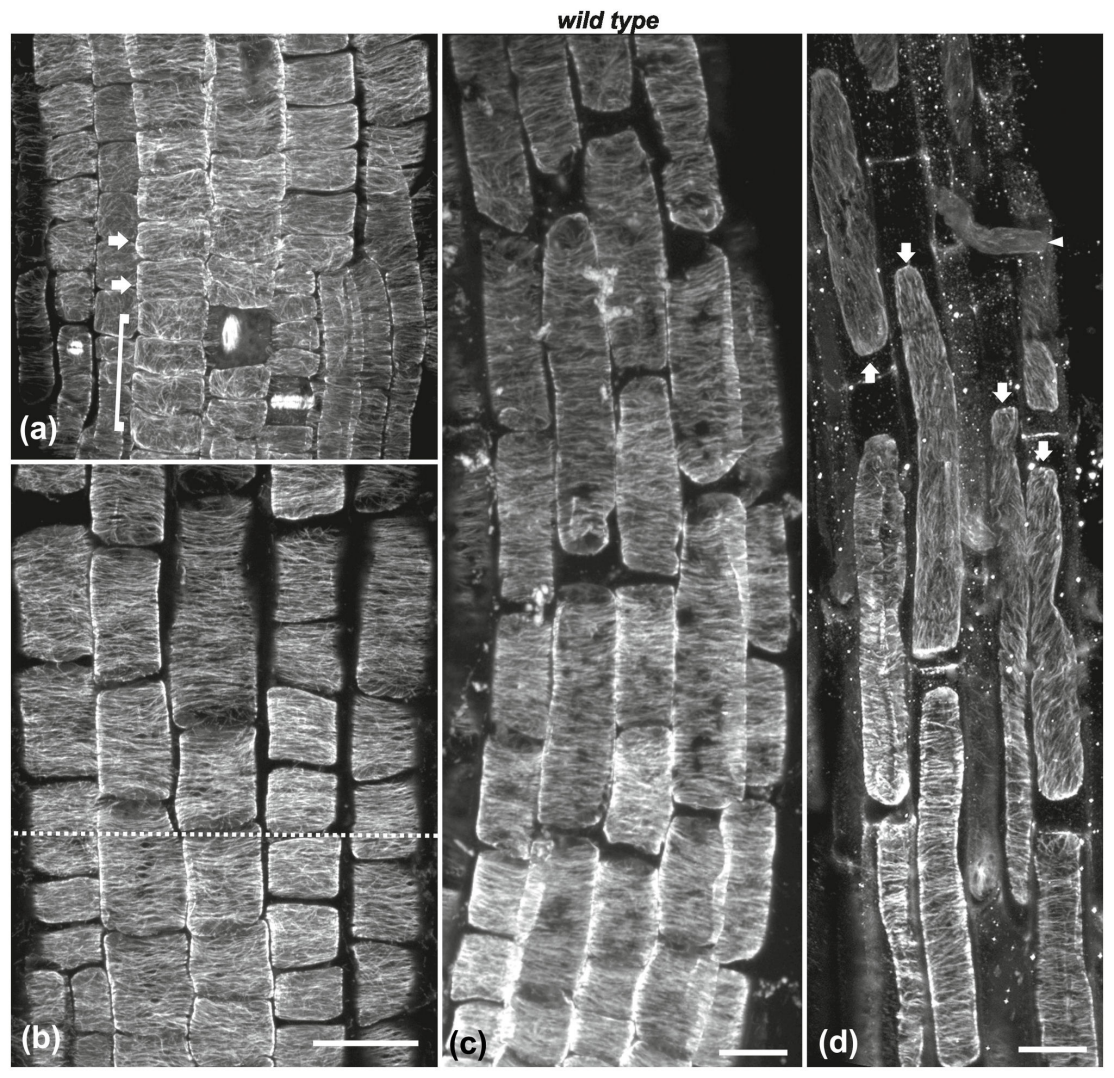
Cortical microtubule orientation at the external cell faces of wild-type roots. Maximum projections of CLSM sections at the transition (a, b) and elongation (b, c) zones. Cortical microtubule orientation in protodermal cells shifts from loosely longitudinal in the meristematic zone (cells included in bracket in a) to transverse in the early transition zone (cells marked by arrows in a). All cells shootward above this shift exhibit transverse microtubule orientation. The dotted line in (b) denotes the boundary between transition (under dotted line) and early elongation (over dotted line) zone. (c) Cortical microtubules are predominantly transverse even in the longest cells of the elongation zone. (d) At the boundary of fast elongation/ growth terminating zones, microtubule orientation shifts from transverse to oblique or longitudinal (arrows). The arrowhead points to an emerging root hair. Scale bars, 20 μm.

In the transition zone, cortical microtubules under the external protodermal cell wall shifted from loosely longitudinal (Figure 3a; included in bracket) to transverse (Figure 3a, arrows). Consequently, transverse microtubule orientation was uniform in every cell of this zone (Figure 2c), as well as of the fast elongation zone (Figures 3b, c, 2c), while this pattern was altered in the cells of the growth terminating zone (Figure 3d, arrows; [35]). Accordingly, transverse orientation of cortical microtubules was established in the meristematic zone and persisted through the transition and fast elongation zones of *A. thaliana* root.

### Inhibition of cellulose biosynthesis resulted in reorientation of cortical microtubules in the fast elongation zone

To address the question whether defective cell wall synthesis may influence the orientation of microtubules in root tips, the pattern of cortical microtubules in *than* heterozygous and *pom2-4* root tips was examined. The function of CesA3 is aberrant in *than* mutants, resulting in reduced cellulose synthesis and plant growth [33]. Heterozygous *than/*+ plants are semi-dwarf (Figure 2j), while *than* homozygous seedlings die soon after germination [33]. Homozygous *CSI1*/*pom-pom2* (*csi1*/*pom2*) seedlings also exhibit reduced growth phenotype (Figure 2j) with decreased cellulose content and defective hypocotyl and root cell elongation [12-14]. 

**Figure 4:**
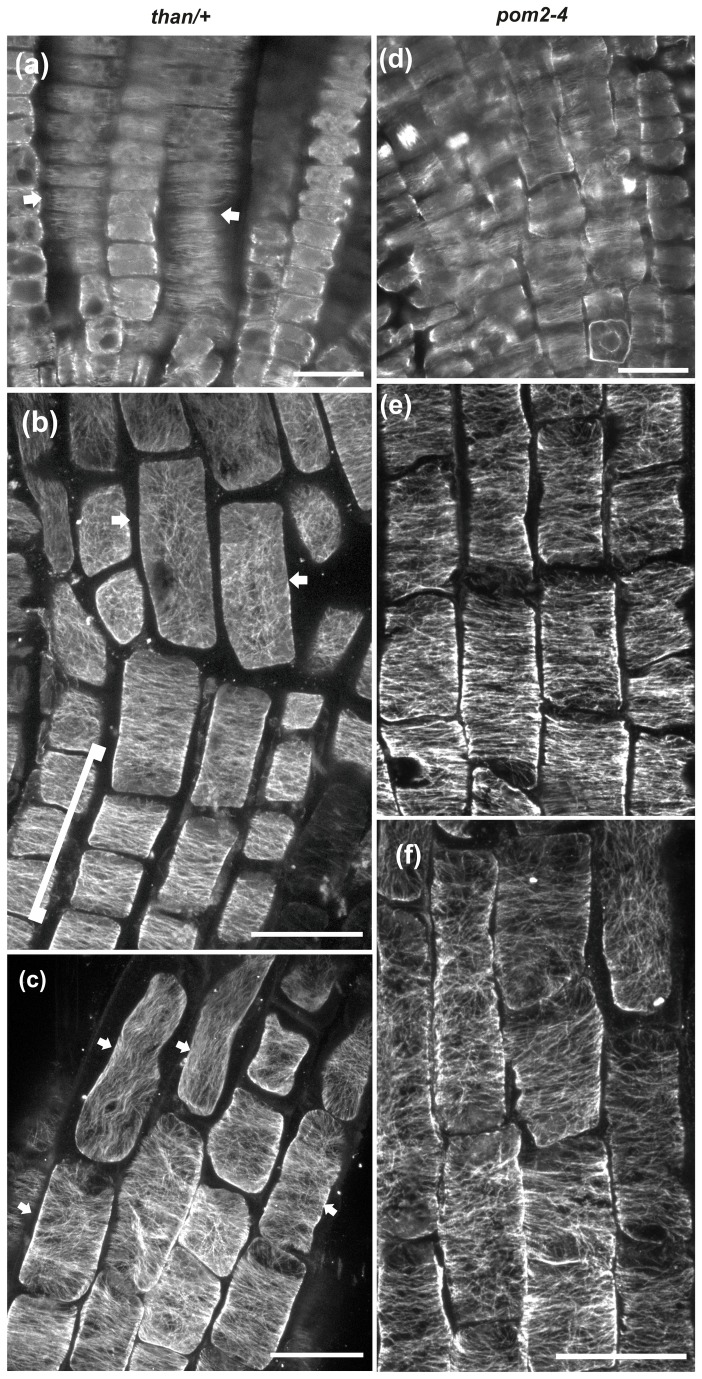
CLSM microtubule images through the tip of *than*/+ (a-c) and *pom2-4* (d-f) roots. Cortical microtubules are mainly transverse (arrow) in the meristematic zone of *than*/+ (a) and *pom2-4* (d). (b, c) Maximum projections of CLSM protodermal/epidermal cell sections in the transition (bracket in b) and elongation zone of *than*/+ root tips. Cortical microtubules are mainly transverse in the transition and early elongation zone but appear reoriented in longer cells of the elongation zone shootward (arrows in b and c). (e, f) Maximum projections of CLSM protodermal cell sections in the early (e) and advanced (f) elongation zone of *pom2-4* root tips. Cortical microtubules are transverse in the shorter cells rootward (e) but appear reoriented in the longer cells shootward (f). Scale bars, 20 μm.

Similarly to wild-type root tips, cortical microtubule orientation was transverse in the meristematic (Figures 4a, pointed by arrows, 4d) and transition (Figures 4b, included by bracket, 4e) zones of *than/+* and *pom2-4* (Figures 2d, e) root tips. Cortical microtubules remained transverse in the epidermal cells of the fast elongation zone located close to the transition zone, while the orientation changed in the elongated cells proximal to the growth terminating zone in both *than/+* (Figures 2e, 4b, c, arrows) and *pom2-4* (Figures 2d, 4f). Measurements of the LEH and of the length of adjacent elongation zone epidermal cells confirmed that the final cell length was significantly reduced in both mutants compared to wild-type seedlings (Figures 2a, b). These results demonstrate that genetic defects in cellulose synthesis restrain cell expansion and modify the orientation of cortical microtubules only in the zone of fast elongation.

**Figure 5:**
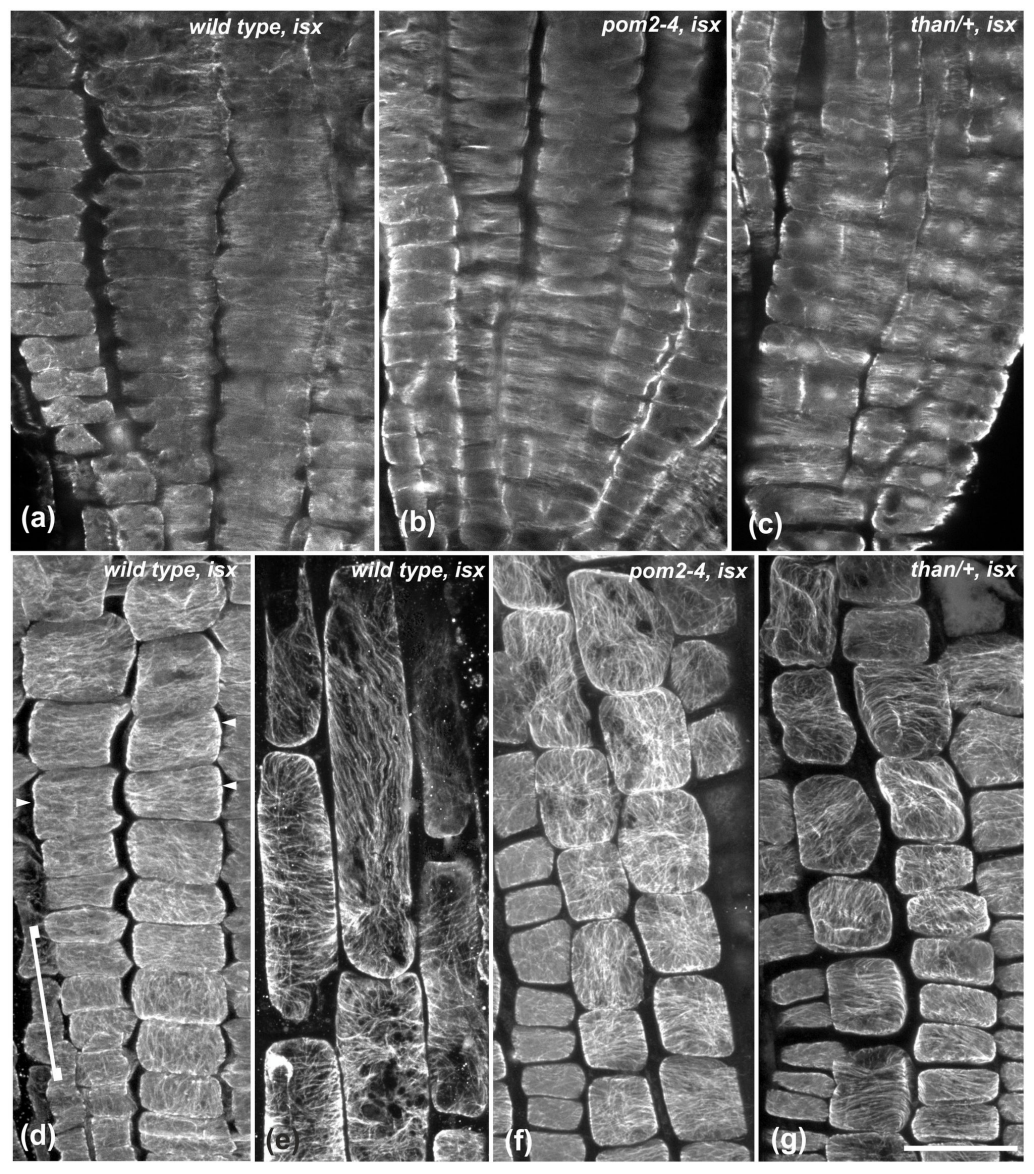
The effect of 4 h treatment with isoxaben (isx) on cortical microtubule orientation. (a-c) Single CLSM sections through the meristematic zone of wild-type (a), *pom2-4* (b) and *than*/+ (c) roots. Cortical microtubules are mainly transverse. (d) Maximum projection of protodermal cell CLSM sections at the meristematic and early transition zone of wild-type root. Loosely longitudinal microtubule orientation in meristematic cells (included in bracket) shifts to transverse in the transition zone cells (arrowheads). (e) Higher magnification of the wild-type elongation zone epidermal cells included in the dotted line frame in Figure S1. Cortical microtubules exhibit various orientations. (f, g) Transition and early elongation zone of *pom2-4* (e) and *than*/+ (f) roots at maximum projections of protodermal/epidermal cell CLSM sections. Cortical microtubules are randomly oriented. Scale bar, 20 μm.

Given that genetic impairment of cellulose synthesis induced the above results, we assessed the effect of chemical inhibition of cellulose synthesis on cortical microtubules. The cellulose synthesis inhibitor isoxaben [40] was applied on wild-type (Figure 2k) and mutant seedlings. Isoxaben treatment for 4-6 h did not exert any effect on the transverse microtubule orientation of the cells in the meristematic (Figure 2f, 5a) and transition (Figures 2f, 5d, arrowheads) zones of wild-type roots. Cortical microtubules remained transverse in the short cells located rootward in the fast elongation zone (Figure S1, arrows), but were reoriented in the elongated epidermal cells located shootward, proximal to the growth terminating zone (Figures 2f, 5e). Isoxaben treatment for 6 h also reduced the length of fast elongation zone cells in wild-type roots, as indicated by measurements of the LEH and of the length of adjacent cells rootward (Figures 2a, b). This also indicates that a biophysical feedback from the cell wall affects the transverse orientation of cortical microtubules. 

The pattern of microtubule organization in *than*/+ and *pom2-4* meristematic zone cells remained transverse when treated with isoxaben (Figures 5b, c), but it was altered in the cells of the transition and fast elongation zones (Figures 5f, g). The extension of microtubule reorientation in the transition zone results from the combinatorial action of genetic defects and chemical inhibition of cellulose synthesis, indicating that intense perturbation of cellulose biosynthesis affected the pattern rootward. This also underlines the interplay between cellulose synthesis and microtubule orientation.

### Inhibition of cell expansion results in cortical microtubule reorientation in the elongation zone

**Figure 6:**
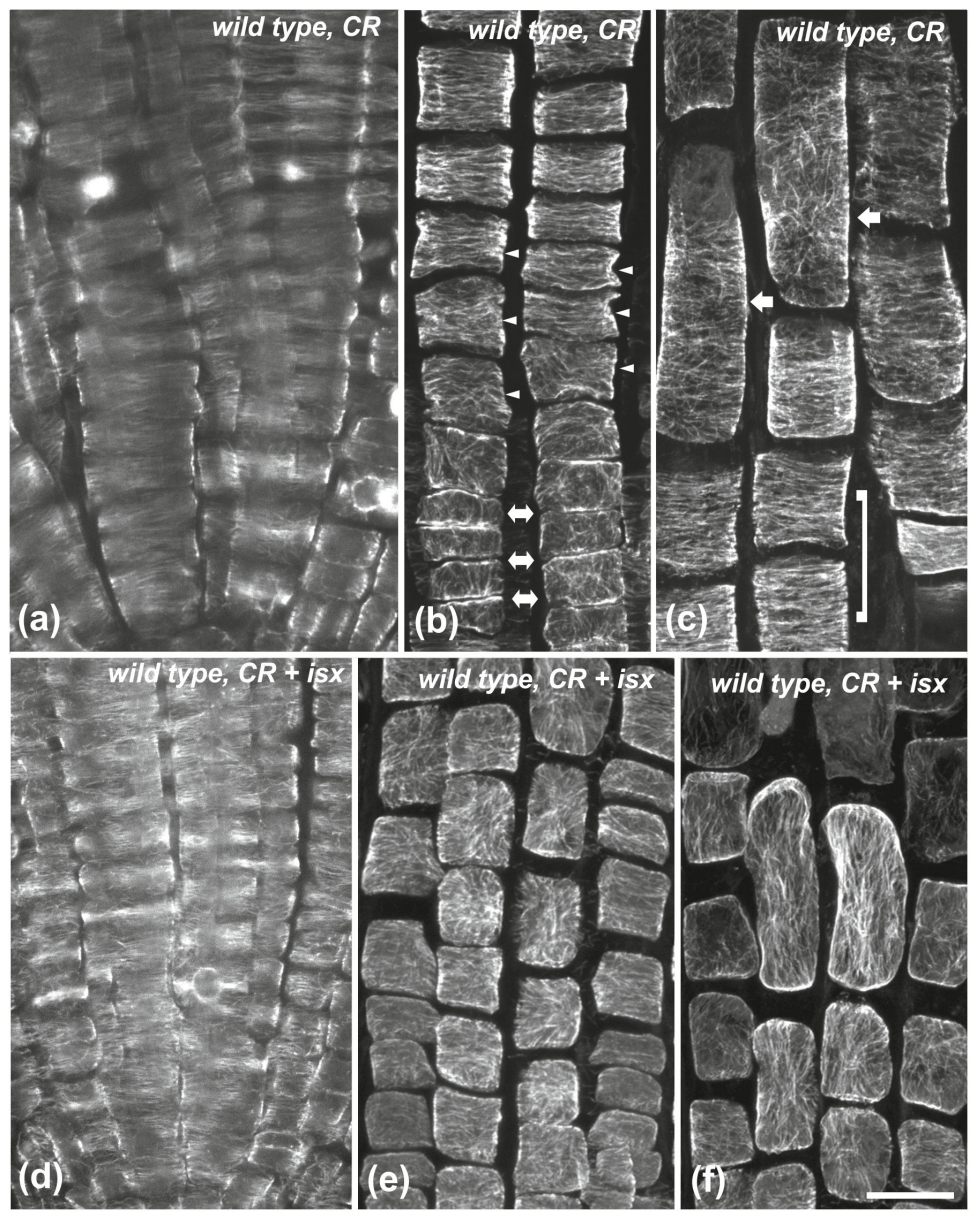
The effect of Congo red (CR) and Congo red + isoxaben (CR+isx) on cortical microtubules of wild-type roots. Treatment for 6 h with Congo red (a-c) and Congo red + isoxaben (d-f), at single CLSM sections (a, d) or maximum projections (b, c, e, f). (a, d) In the meristematic zone cortical microtubules appear mainly transverse. (b) External protodermal cell face at the meristematic-transition zone boundary. Cortical microtubules, though loosely longitudinal in the meristematic zone (double arrows), shift to transverse in the transition zone (arrowheads). (c) In the elongation zone, cortical microtubules appear transverse in the shorter protodermal/epidermal cells rootward (included by bracket) but they appear reoriented in the longer cells shootward (arrows). After the combinatorial treatment, cortical microtubules exhibit random orientation in the transition (e) and elongation (f) zones. Scale bar, 20 μm.

Since impaired cellulose synthesis reduced cell length and concomitantly induced cortical microtubule reorientation, the effects of cell growth inhibitors on the orientation of microtubules were investigated (Figure 2k). The cellulose-binding stain Congo red inhibits cell expansion without affecting cellulose synthesis, by potentially uncoupling cellulose polymerization from its crystallization into microfibrils [41,42]. Similarly to isoxaben, Congo red apparently decreased cell length in the fast elongation zone (Figures 2a, b). Cortical microtubule orientation remained transverse in the meristematic and transition zones of wild-type root tips treated with Congo red for 6 h (Figures 2g, 6a, b, arrowheads). However, in the fast elongation zone this pattern changed in the cells located shootward, close to the growth terminating zone (Figures 2g, 6c, arrows), albeit it was still transverse in the cells located rootward, close to the transition zone (Figure 6c; included in bracket). 

**Figure 7:**
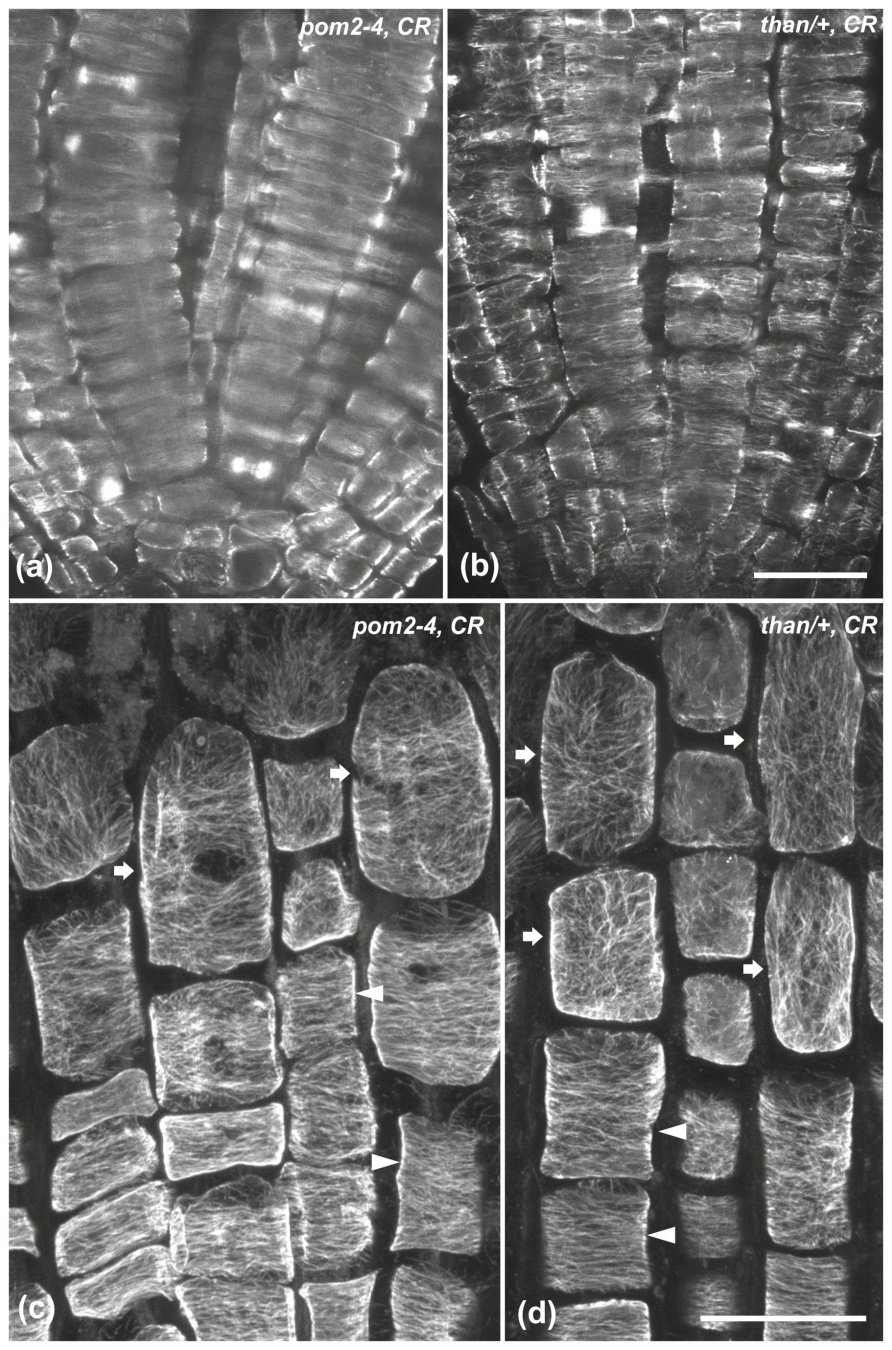
The effect of Congo red (CR) on the microtubules of *pom2-4* and *than/*+ root tips. Treatment for 6 h on *pom2-4* (a, c) and *than*/+ (b, d) root tips, at single CLSM sections (a, b) or maximum projections (c, d). In both mutants, cortical microtubules appear mainly transverse in the meristematic zone (a, b). In the transition zone (arrowheads in c, d), protodermal cells exhibit transverse microtubules, while in the elongation zone (arrows in c, d) cortical microtubules are randomly oriented. Scale bars, 20 μm.

As expected, the meristematic cells of wild-type roots treated simultaneously with Congo red and isoxaben for 6 h had transverse cortical microtubules (Figure 6d). However, the orientation was modified in the cells of the transition and fast elongation zones (Figures 6e, f). In *pom2-4* and *than*/+ root tips treated with Congo red cortical microtubules remained transverse in the meristematic (Figures 7a, b) and transition (Figures 7c, d, arrowheads) zones but they were reoriented in the fast elongation zone (Figures 7c, d, arrows). 

**Figure 8:**
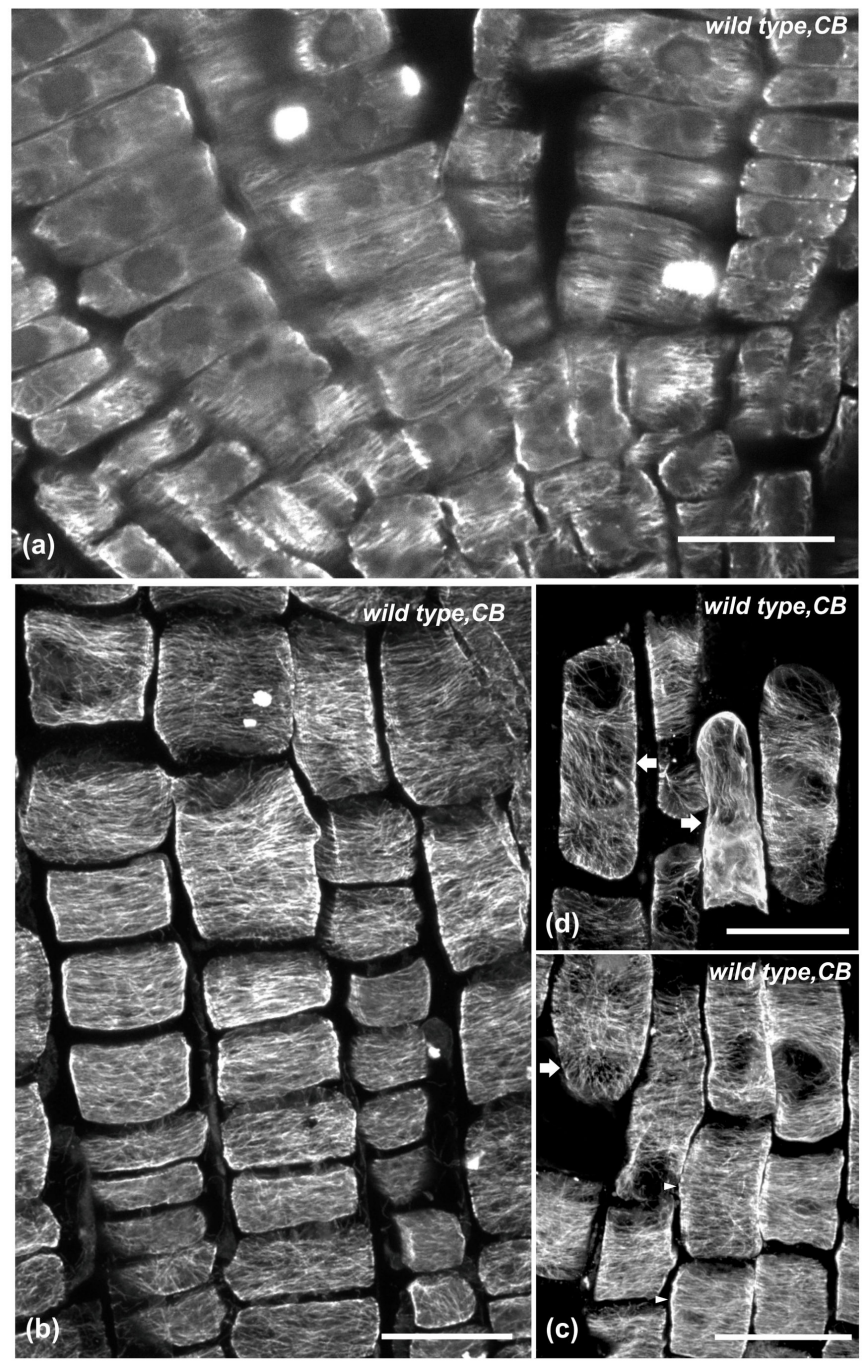
The effect of 6 h cytochalasin-B (CB) treatment on cortical microtubules of wild-type root tips. Single CLSM section (a) or maximum projections (b-d). Cortical microtubules are mainly transverse in the meristematic (a), transition (b) and early elongation (arrowheads in c) zone. In the longer epidermal cells of the elongation zone (arrows in c and d), shootward, microtubules are randomly oriented. Scale bars, 20 μm.

**Figure 9:**
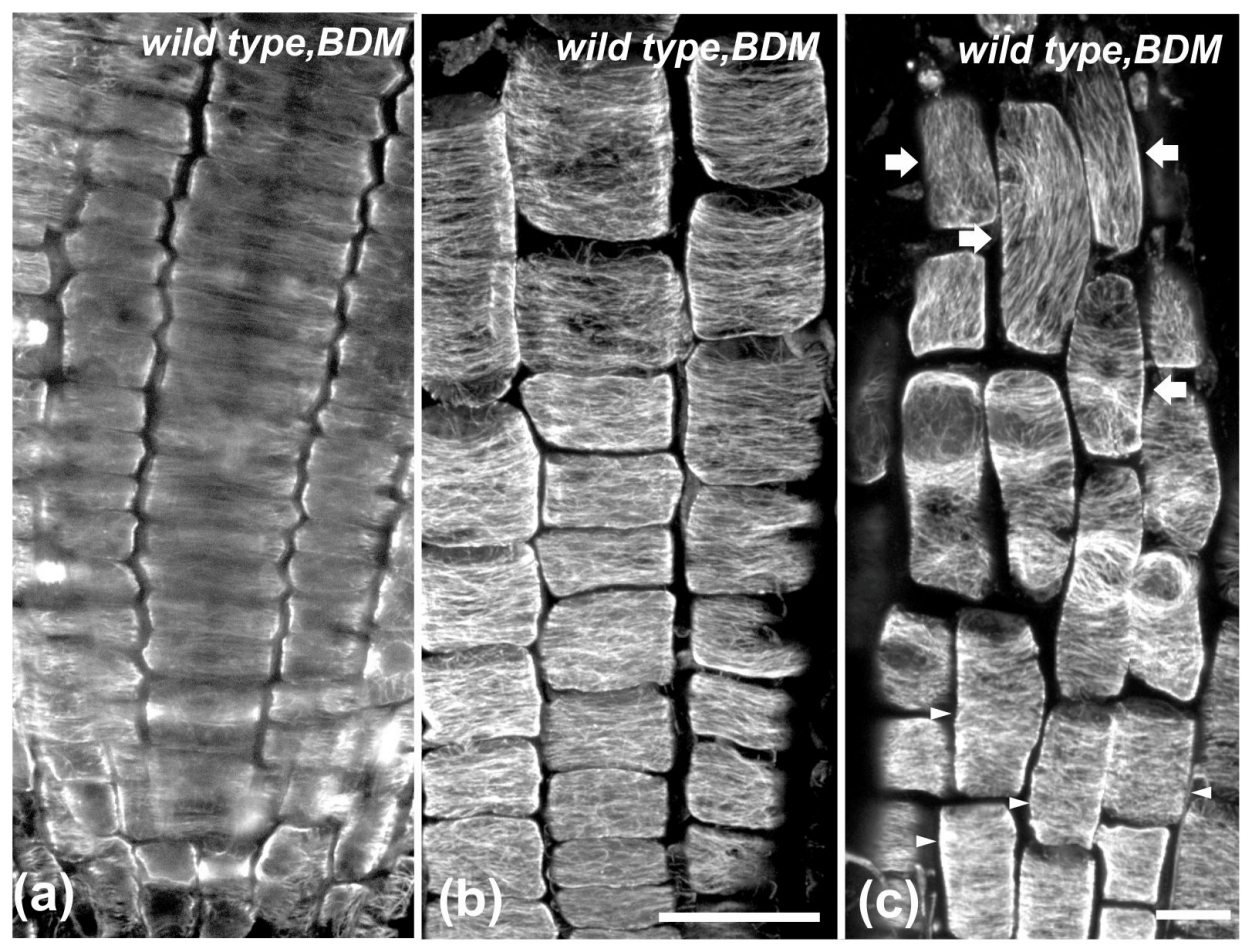
The effect of 6 h BDM treatment on cortical microtubules of wild-type root tips. Single CLSM section (a) or maximum projections (b, c). Cortical microtubules appear mainly transverse in the meristematic (a), transition (b) and early elongation (arrowheads in c) zone, while they appear reoriented in the longer epidermal cells of the elongation zone (arrows in c). Scale bars, 20 μm.

Apart from Congo red, anti-actomyosin drugs affecting cell elongation [43,44] were also applied. Wild-type seedlings were treated for 6 h with the actin-depolymerizing drug cytochalasin-B or the myosin inhibitor BDM (Figure 2k). In roots of these seedlings, as in wild-type seedlings treated with isoxaben or Congo red, the LEH and the length of the adjacent epidermal cells rootward were reduced compared to untreated seedlings (Figures 2a, b). Cortical microtubules were transversely oriented in cells of the meristematic (Figures 8a, 9a) and transition zones (Figures 8b, 9b), as well as in cells of the fast elongation zone located rootward (Figures 8c, 9c, arrowheads). Nevertheless, microtubules were reoriented in elongated cells located shootward in the fast elongation zone, close to the growth terminating zone (Figures 8c, d, 9c, arrows). Taken together, these results demonstrate that the severity of chemical inhibition on cell expansion affects transverse microtubule orientation in a cell position-dependent manner (Figures 2g-i).

### Mechanical impedance reduced cell length and induced microtubule reorientation in the fast elongation zone

To further examine how inhibition of cell expansion or alteration of biophysical feedback could be associated with microtubule reorientation, roots were subjected to mechanical impedance by growing in soil. As roots penetrate into the soil, they must overcome its physical resistance [45,46]. This approach can be applied to unravel the effect of mechanical forces on root growth and microtubule orientation. The primary root of wild-type seedlings grown in soil was shorter compared to that of seedlings grown in Petri dishes (Figures 2k, S2). The distance between the quiescent center and the first cell forming a root hair was quite variable. This heterogeneity is most likely due to soil moisture, density or confinement. Hence, as soil strength increases, the fast elongation zone is plausible to become shorter. Likewise, the final cell length decreased (50-80 μm), compared to roots grown in Petri dishes (110-150 μm). 

**Figure 10:**
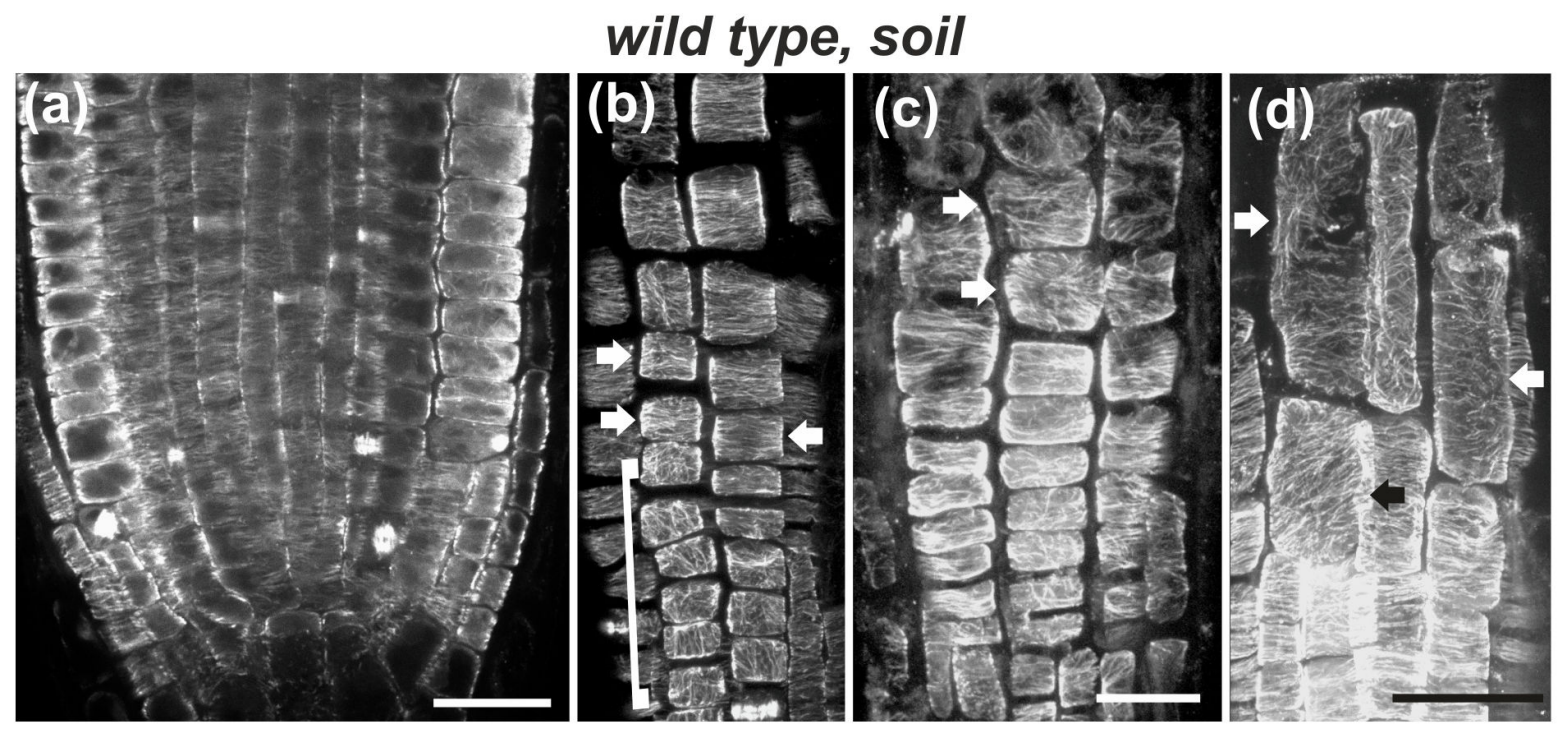
The effect of penetration in soil on cortical microtubules of wild-type root tips. Single CLSM section (a) or maximum projections of CLSM sections (b-d). (a) Cortical microtubules are transversely oriented in the meristematic zone. (b) In protodermal cells, loosely longitudinal orientation (cells included in bracket) shifts to transverse (arrows) in the transition zone. (c) In this extremely short root tip (see root tip in Figure S2) cortical microtubules are not transverse even in the shorter protodermal/epidermal cells of the elongation zone (arrows). (d) In this root tip, cortical microtubules exhibit random orientation in the longer epidermal cells of the elongation zone (arrows). Scale bars, 20 μm.

The orientation of cortical microtubules in the meristematic and transition zones of soil-grown roots was transverse (Figures 10a, b, arrows), similar to wild-type roots grown in Petri dishes (cf. Figures 1b, 3a). However, microtubule orientation was altered in the fast elongation zone, depending on cell size reduction. In roots with short cells, cortical microtubules exhibited random orientation throughout the fast elongation zone (Figure 10c, arrows). In roots with longer elongation zone cells, cortical microtubules appeared transverse in the cells located rootward, whereas their orientation became random in the elongated cells located shootward (Figure 10d, arrows). These results show that inhibition of cell elongation by mechanical impedance perturbs the organization of cortical microtubules in the fast elongation zone without affecting the transverse orientation in the meristematic and transition zones. 

## Discussion

### Transverse orientation of cortical microtubules is established in the meristematic root zone

This study provides conclusive evidence that cortical microtubule orientation in the meristematic zone of *A. thaliana* root is predominantly transverse, perpendicular to the root axis. However, the orientation of cortical microtubules is often determined by the specific stage of the cell cycle and/or the exact position of each individual cell. Accordingly, the variability in microtubule orientation reported previously [1,31,32] may be due to the presence of post-cytokinetic cells with randomly oriented microtubules, cells undergoing formative divisions, or to the microtubules at the external protodermal cell face. 

Microtubule organization in distinct patterns at different cell faces is typical in protodermal/epidermal cells of stems and leaves [11,47-52]. In root protodermal cells such a dual pattern could be attributed to the local accumulation of CLASP (Cytoplasmic Linker Protein-Associated Protein) that allows microtubules to grow around sharp cell edges and prevents depolymerization [39]. Cell edges of post-cytokinetic cells also accumulate components of the γ-tubulin complex [53], involved in microtubule nucleation. These complexes may also participate in the formation of the specific microtubule pattern in different edges of protodermal cells. As cell divisions cease in the transition zone, these protein complexes are no further deposited at cell edges and thereby cortical microtubules under the external wall become transverse. Therefore, transverse orientation of cortical microtubules is established in the meristematic cells and is perpetuated through the transition and fast elongation zones until the growth terminating zone (Table 1).

**Table 1:** Presentation of the differential responsiveness of microtubule orientation to genetic, chemical and mechanical cues in the developmental zones of *A.thaliana* root.

	**Wild type (WT) control**	**WT: isx**	**WT: CR**	**WT: CR + isx**	**WT: CB**	**WT: BDM**	**WT: Soil**	***than/*+ control**	***than/*+: isx**	***than/*+: CR**	***pom2-4* control**	***pom2-4*: isx**	***pom2-4*: CR**
**Growth terminating zone**	**R**	**R**	**R**	**R**	**R**	**R**	**R**	**R**	**R**	**R**	**R**	**R**	**R**
**Fast elongation zone**	**T**	**R**	**R**	**R**	**R**	**R**	**R**	**R**	**R**	**R**	**R**	**R**	**R**
**Transition zone**	**T**	**T**	**T**	**R**	**T**	**T**	**T**	**T**	**R**	**T**	**T**	**R**	**T**
**Meristematic zone**	**T**	**T**	**T**	**T**	**T**	**T**	**T**	**T**	**T**	**T**	**T**	**T**	**T**

**T**: Transverse orientation of cortical microtubules, **R**: Random or longitudinal orientation of cortical microtubules, **isx**: Isoxaben, **CR**: Congo Red, CB: Cytochalasin-B, **BDM**: 2,3-butanedione monoxime, ***than***: *CesA3* mutant allele, ***pom2-4***: *CSI1* mutant allele

Our observations on cortical microtubules and those on cellulose microfibrils [1] support that there is an overall match in orientation between the above components in the meristematic root zone. Cellulose microfibrils are transverse in the inner wall of protodermal cells but not in the external wall [1]. Likewise, cortical microtubules are transversely oriented at the inner periclinal protodermal cell faces, but not at the external cell face. Apart from protodermal cells in the meristematic zone, cortical microtubules and cellulose microfibrils share the same transverse orientation in all cells of the meristematic, transition and fast elongation zones. Consequently, consistent co-alignment between cortical microtubules and cellulose microfibrils occurs along the root endowing a uniform mechanical structure [54] that allows the root to grow strictly axially like a cable. 

### Transverse orientation of cortical microtubules in the fast elongation zone requires undisturbed cell expansion

Fisher and Cyr [22] provided the first evidence that cortical microtubule orientation depends on a biophysical feedback from the cell wall. Since then, studies with CesA inhibitors [23,25,26] and *CesA* mutants [24,25] supported that transverse cortical microtubule orientation depends on undisturbed cellulose synthesis. Microtubule organization is particularly supported to be directly influenced by CesA function [25]. In the present study, however, cortical microtubule reorientation in the fast elongation zone (Table 1) appears to be due to inhibition of cell elongation and not to inhibition of CesA activity *per se*. Although *than* and *pom2-4* are both cellulose-deficient mutants, *pom2-4* is a mutant of CSI1 [14] and not of CesAs. Therefore, microtubule reorientation in *pom2-4* root (Table 1) is most likely not due to malfunction of cellulose synthase complexes. In addition, if mutation of *CesA3* in *than* could directly induce cortical microtubule reorientation, this should also occur in the meristematic and transition zones, as *CesA3* is expressed in these zones [12]. However, in *than* roots cortical microtubules in the latter zones remained transverse (Table 1). Accordingly, a direct causal relationship between malfunction of cellulose synthase complexes and microtubule orientation cannot be supported. 

A common feature between *than* and *pom2-4* mutants, and wild-type roots treated with isoxaben, was the decreased cell length (Figures 2a, b). In normally growing wild-type roots, cortical microtubule reorientation is evident close to the growth terminating zone, as cell elongation attenuates [1,35]. Consequently, cortical microtubule reorientation in the fast elongation zone of the mutants or isoxaben-treated wild-type roots (Table 1) could result from a premature cessation of cell expansion, due to cellulose shortage, as shown for hypocotyl cells [55]. This view is in agreement with the suggestion that in ethylene-treated roots inhibition of cell elongation precedes microtubule reorientation [35]. In epicotyl segments of azuki bean, exogenous application of auxin under aerobic conditions stimulated the elongation of epidermal cells and caused the reorientation of microtubules from longitudinal to transverse. Though the orientation of microtubules still changed upon auxin application under anaerobic conditions, cell elongation was nevertheless inhibited [56]. However, according to the Cholodny-Went theory, the root and shoot cells differ considerably in their responsiveness to auxin, as the accumulation of auxin in shoots induces cell elongation whereas in the root auxin inhibits cell elongation. On the basis of our results we postulate that cortical microtubules in the shoot and root could respond differently to suppression of cell elongation, albeit the influence of anoxia on microtubule patterning remains elusive as yet.

Congo red and anti-actomyosin drugs have been shown to decrease cell expansion without affecting cellulose synthesis [42-44,57]. The application of chemical compounds inhibiting cell expansion led to reorientation of cortical microtubules in the fast elongation zone of wild-type roots (Table 1). The results obtained by cytochalasin-B treatment are notably similar to those observed in *Zea mays* roots treated with another anti-actin drug, latrunculin-B [57]. Mechanical impedance of cell expansion due to growth in soil also resulted in microtubule reorientation in the fast elongation zone. Interestingly, this natural suppression of cell elongation induced results similar to chemical treatments without affecting cellulose synthase complexes. Taken together, our data imply that transverse microtubule orientation in the fast elongation zone is altered due to perturbed cell expansion rather than malfunction of cellulose synthase complexes. Inhibition of cell elongation, irrespective of its cause, provides the protoplast with a biophysical feedback from the cell wall, resulting in cortical microtubule reorientation. However, the possibility that biochemical parameters or that a combinatorial biophysical and biochemical mechanism may also influence the reorientation of microtubules cannot be ruled out.

### Transverse microtubule orientation is more stable in meristematic and transition zone cells than in rapidly elongating cells

Although in both mutants and under several experimental manipulations cortical microtubule orientation was altered in the fast elongation zone, it remained unaffected in the meristematic zone (Table 1). This remarkable difference could be attributed to the specific developmental course and cell expansion pattern in each zone. In general, the slow but steady anisotropic expansion of meristematic cells has been overlooked [58]. Nevertheless, meristematic cells expand anisotropically after completion of cytokinesis, increasing their length parallel to the root axis (Figure S3) in order to divide again. As cell divisions were not inhibited in the mutants or in any of the experimental applications in this study, premitotic cell expansion was not significantly suppressed, resulting in perseverance of transverse cortical microtubule orientation in meristematic cells. 

Cells of the transition zone are characterized by slow anisotropic expansion, as they prepare for rapid elongation once they enter the fast elongation zone [37]. It seems that this slow expansion sustains the transverse pattern of cortical microtubules, already established in the meristematic zone. This was altered only under the combinatorial application of Congo red and isoxaben on wild-type roots, or the treatment of *than/+* and *pom2-4* mutants with isoxaben (Table 1).

On the contrary, cortical microtubule orientation was affected in the fast elongation zone in both mutants and under all the experimental conditions applied herein (Table 1), in combination with suppressed cell expansion. Several factors could hinder rapid cell elongation. Rapidly elongating cells most probably require a high rate of cellulose supply. In cellulose-deficient mutants or isoxaben-treated seedlings this supply was reduced. Congo red disturbs cellulose conformation, hindering fast cell elongation and ultimately leading to cortical microtubule reorientation. Anti-actomyosin drugs may inhibit cytoplasmic streaming in the highly vacuolated cells of the fast elongation zone and/or limit the motility of CesA-containing dictyosomes and the transport of CesA-baring vesicles to the plasma membrane [59], suppressing thus cell elongation. Rapid cell expansion in the fast elongation zone depends on mechanisms and processes different from those regulating the slow cell growth in the meristematic [58] and transition [37] zones. In fact, cells in the elongation zone have different cell wall properties from those of the meristematic and transition zones, and are influenced by a particular hormonal milieu [37]. These characteristics imply that cells in each zone represent different developmental stages and most likely possess distinct biophysical traits, implicated in anisotropic cell expansion. The stability or susceptibility of cortical microtubule orientation seems to be an internal trait of this process. 

### Concluding remarks

Herein the patterns of cortical microtubule orientation in the developmental zones of *A. thaliana* root were assessed. Transverse orientation of cortical microtubules is established in the meristematic zone, to support moderate premitotic anisotropic cell expansion. This pattern perseveres in cellulose deficient mutants and under chemical or mechanical treatments. In the fast elongation zone, microtubule orientation is rather responsive to fluctuations of cell elongation rate than directly depending on CesA activity, due to the particular biophysical and/or biochemical properties of rapidly elongating cells. Further analysis is required to elucidate the biological and mechanical factors, responsible for the establishment and stabilization of transverse cortical microtubule orientation in meristematic cells.

## Supporting Information

Figure S1 **Cortical microtubules in the elongation zone of an isoxaben-treated wild-type root.** Maximum projection of CLSM sections through the elongation zone epidermis. Cortical microtubules appear transverse in the shorter cells rootward (arrows) but exhibit various orientations in the longer cells shootward, included in the dotted line frame. Higher magnification of this frame is depicted in Figure 7e. Scale bar, 20 μm.
(TIF)

Figure S2 **A phenotype of wild-type root tip grown in soil.** Note that cell length is decreased and root hairs (arrow) emerge close to the root tip. Scale bar, 20 μm.
(TIF)

Figure S3 **Dividing protodermal cells in wild-type root meristem at single CLSM section.** Note that the preprophase (a), telophase (b) and cytokinetic (c) cells are longer than the adjacent interphase cells. Scale bar, 10 μm.
(TIF)
