# Supplementary figures and images for: Differential Responsiveness of Cortical Microtubule Orientation to Suppression of Cell Expansion among the Developmental Zones of Arabidopsis thaliana Root Apex
Source: PLoS One. 2013 Dec 4;8(12):e82442. doi: 10.1371/journal.pone.0082442 (PMC3853581; doi:10.1371/journal.pone.0082442)

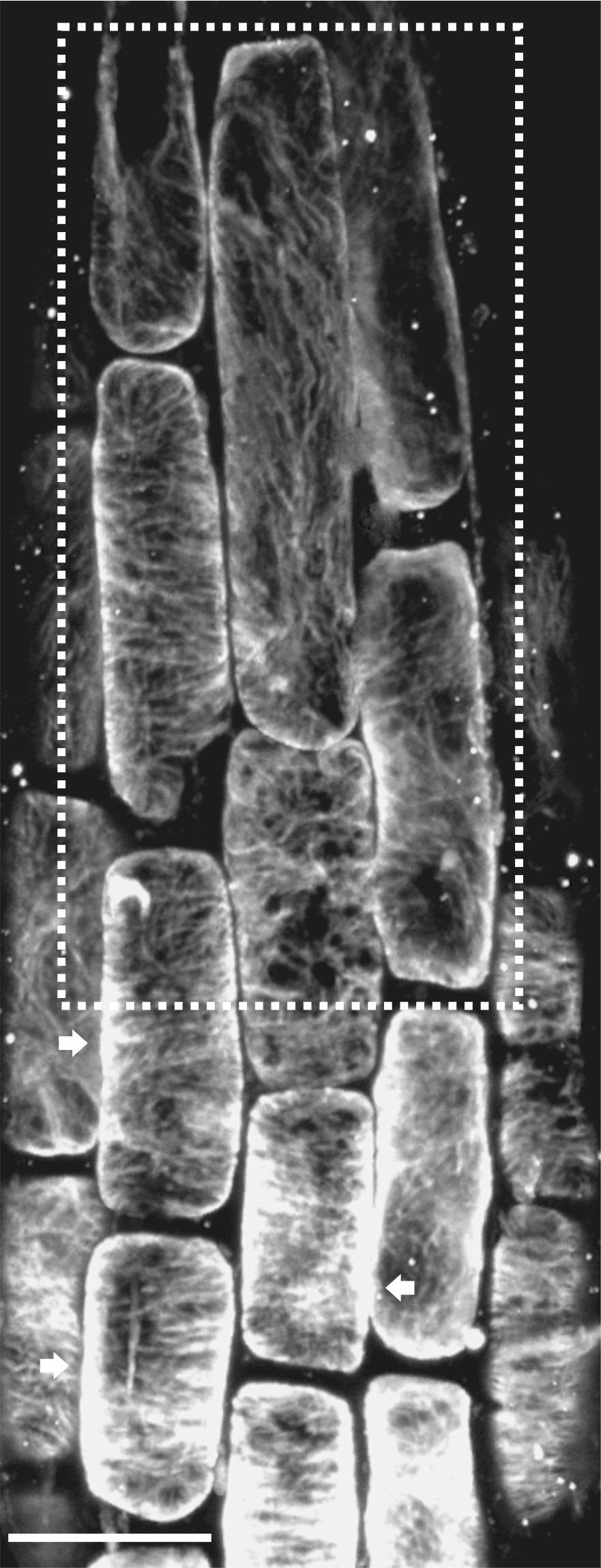

Supplement: Figure S1 — Cortical microtubules in the elongation zone of an isoxaben-treated wild-type root. Maximum projection of CLSM sections through the elongation zone epidermis. Cortical microtubules appear transverse in the shorter cells rootward (arrows) but exhibit various orientations in the longer cells shootward, included in the dotted line frame. Higher magnification of this frame is depicted in Figure 7e. Scale bar, 20 μm. (TIF) [file pone.0082442.s001.tif]

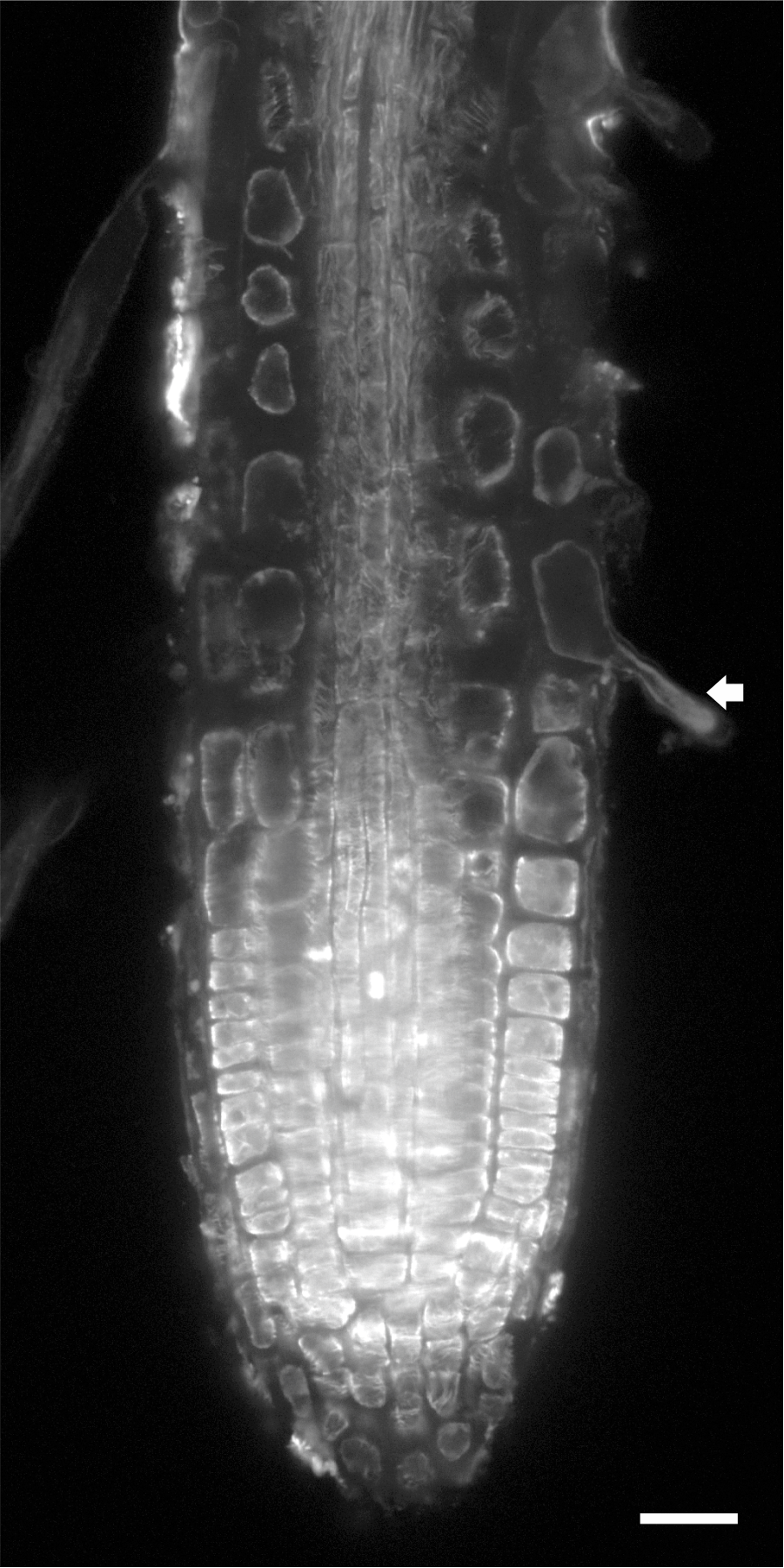

Supplement: Figure S2 — A phenotype of wild-type root tip grown in soil. Note that cell length is decreased and root hairs (arrow) emerge close to the root tip. Scale bar, 20 μm. (TIF) [file pone.0082442.s002.tif]

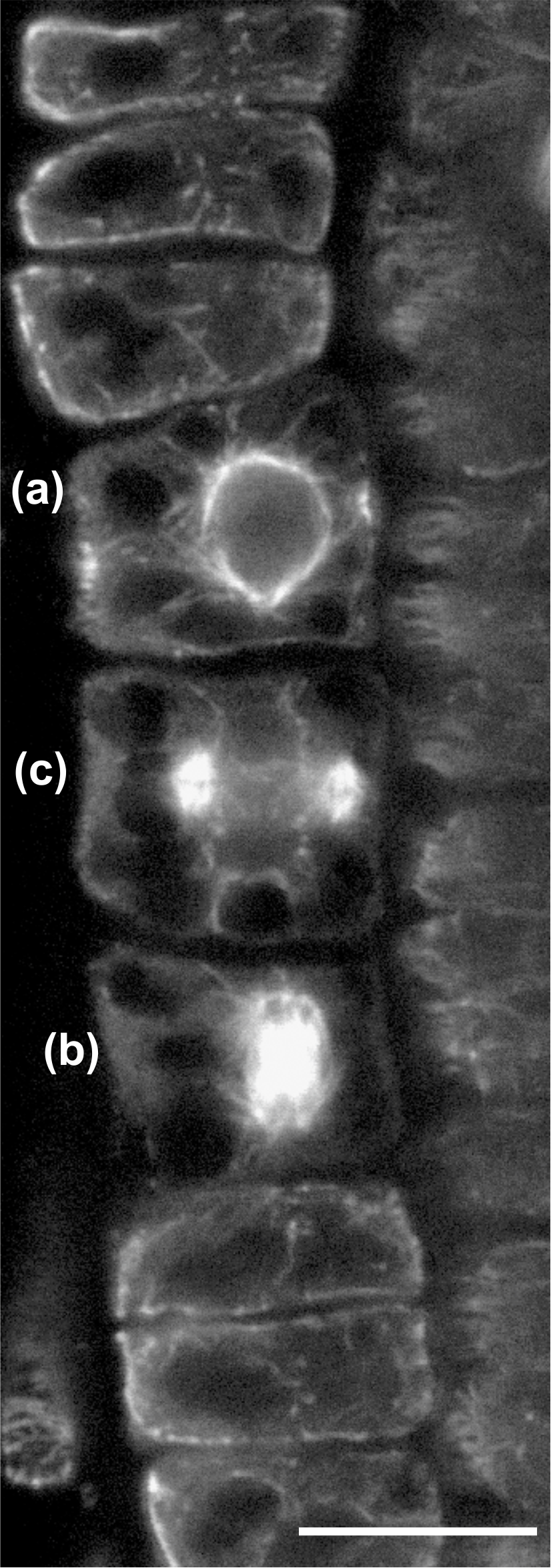

Supplement: Figure S3 — Dividing protodermal cells in wild-type root meristem at single CLSM section. Note that the preprophase (a), telophase (b) and cytokinetic (c) cells are longer than the adjacent interphase cells. Scale bar, 10 μm. (TIF) [file pone.0082442.s003.tif]
